# Physiological deterioration prior to in-hospital cardiac arrest: What does the National Early Warning Score-2 miss?

**DOI:** 10.1016/j.resplu.2024.100788

**Published:** 2024-09-30

**Authors:** Sherif Gonem, Daniella Draicchio, Ayad Mohamed, Sally Wood, Kelly Shiel, Steve Briggs, Tricia M McKeever, Dominick Shaw

**Affiliations:** a Department of Respiratory Medicine, Nottingham University Hospitals NHS Trust, Nottingham, UK; b NIHR Nottingham Biomedical Research Centre, School of Medicine, University of Nottingham, Nottingham, UK; c Recognise and Rescue, Nottingham University Hospitals NHS Trust, Nottingham, UK; d Digital and Information, Nottingham University Hospitals NHS Trust, Nottingham, UK; e NIHR Leicester Biomedical Research Centre, School of Medicine, University of Leicester, Leicester, UK

**Keywords:** Cardiac arrest, Clinical deterioration, Rapid response team, Early warning score, Vital signs

## Abstract

**Aim:**

To determine the frequency with which the National Early Warning Score-2 (NEWS-2) fails to detect physiological deterioration preceding in-hospital cardiac arrest (IHCA).

**Methods:**

We conducted a retrospective observational study of all adult patients (age ≥ 18) who had suffered an IHCA between 1st July 2019 and 31st December 2021 in two large acute hospitals located in an urban centre (Nottingham, UK). Clinical observations and case notes were examined for the period leading up to IHCA events to determine if there was evidence of physiological deterioration which warranted an urgent patient assessment, whether NEWS-2 was triggered, and whether an urgent assessment actually took place.

**Results:**

Urgent assessment was indicated in the lead-up to 126/374 (33.7 %) IHCA cases, and NEWS-2 failed to trigger in 20 of these cases (15.9 %). An urgent assessment took place in 89/106 (84.0 %) cases where NEWS-2 was triggered, and 13/20 (65.0 %) cases where NEWS-2 was not triggered, with the difference in proportions being statistically significant (p = 0.048). Half of cases in which NEWS-2 missed a physiological deterioration were related to a new or rising oxygen requirement.

**Conclusions:**

A significant proportion of IHCA events are preceded by clinically important abnormalities in vital signs which are not detected by NEWS-2. This may be a causative factor in some failure-to-rescue events.

## Introduction

In-hospital cardiac arrest (IHCA) is often preceded by a period of physiological deterioration, which is detectable through changes in vital signs^1^. The National Early Warning Score-2 (NEWS-2) is a widely used and well-validated early warning score,^2^, ^3^ but can miss some cases of clinically important deterioration,^4^ as well as having a relatively high false-alarm rate^5^.

We conducted a retrospective observational study of all IHCA events occurring at Nottingham University Hospitals NHS Trust (NUH) between 1st July 2019 and 31st December 2021, in order to determine:

i) the proportion of IHCA events which were preceded by a clinically important deterioration in one or more vital signs, meaning that an urgent patient assessment was indicated.
ii) the proportion of these cases in which NEWS-2 was triggered, and in which an urgent assessment actually took place in the lead-up to IHCA.

## Materials and methods

### Setting

NUH comprises two large acute hospitals located in the East Midlands region of the UK, with approximately 85,000 emergency admissions per year. Clinical observations are recorded electronically using the Nervecentre system, which automatically calculates NEWS-2. The mandated frequency of observations ranges from every 30 min to 12-hourly, depending on the previous NEWS-2 score, with observations mandated at least every 4 h during the first 24 h after admission. NUH has an established rapid-response system known as Hospital 24. This comprises a team of nurse coordinators who continuously monitor NEWS-2, and determine the need to trigger an urgent patient assessment by doctors working on the wards during the day or by the on-call medical team out-of-hours. These triggers can be over-ridden at the discretion of the registered nurse looking after the patient.

### Data collection

We defined IHCA as a resuscitation event, commencing in-hospital, where an individual received chest compression(s) and/or defibrillation and was attended by the hospital-based resuscitation team in response to a cardiac arrest call^6^. A register of IHCA events is kept at NUH for the purposes of audit and service evaluation by the Resuscitation Department. The dates, times and patient identifiers of IHCA events occurring between 1st July 2019 and 31st December 2021 in adult patients (age ≥ 18) were obtained from the register. This included the first three waves of the Covid-19 pandemic in the UK. Clinical observations and NEWS-2 scores for the relevant admission episodes were extracted. IHCA events were included in the analysed dataset if the case notes were available, at least one complete observation set was recorded on Nervecentre during the 24 h prior to the event, and no other IHCA had occurred during the previous 24 h. Critical Care areas at NUH use paper charts to record vital signs, so IHCA events occurring in these areas were not included.

### Case note review

i) IHCA cases were retrospectively reviewed by three expert clinicians, who were all at Consultant (SG) or Specialty Registrar level (DD, AM), to determine if an urgent patient assessment was clinically indicated in the 24-hour period preceding IHCA, according to the clinical judgement of the reviewing clinician. This was based on the recorded clinical observations but without reference to the NEWS-2 score. Deteriorations in observations occurring immediately prior to the IHCA (within 15 min of the event) were considered to be part of the IHCA event itself. Retrospective case review was carried out independently by the three clinicians, with the majority view used in cases of disagreement.
ii) For cases in which an urgent patient assessment was deemed to be indicated, the calculated NEWS-2 score and medical case notes were consulted by a single reviewer (SG) to determine (a) if NEWS-2 reached one of the standard escalation triggers (total score ≥ 5 and/or single observation scoring 3) during the period of deterioration leading up to the IHCA event, and (b) if an urgent patient assessment had taken place during the period of deterioration.
iii) Cases in which NEWS-2 did not trigger despite an urgent assessment being indicated were labelled as “NEWS-2 miss” cases. These were inspected by a single reviewer (SG) to determine the predominant abnormality in vital signs.

### Statistical analysis

Statistical analysis was carried out using IBM SPSS V28.0 with the threshold for statistical significance set at p < 0.05. The Chi-squared test was used to determine whether there was a significant relationship between NEWS-2 being triggered and an urgent patient assessment taking place, where such an assessment was indicated.

### Ethical approval

The project was approved by the Nottingham 1 Research Ethics Committee (20/EM/0064) and the Confidentiality Advisory Group (20/CAG/0034). The analysis presented in this manuscript was approved as an amendment to the study “Detecting clinical deterioration in respiratory hospital patients using machine learning” on 6th October 2022. Study procedures were followed in accordance with the ethical standards of the responsible committee on human experimentation (regional) and the Helsinki Declaration of 1975.

## Results

399 IHCA events occurred during the study period (1st July 2019 to 31st December 2021). 374 events were included in the analysis, with 18 cases excluded due to no complete observation sets being recorded in the previous 24 h, 5 cases excluded due to another IHCA occurring within the previous 24 h, and 2 cases excluded as the notes were not available for review. The 374 analysed IHCA events occurred across 369 admission episodes and the same number of individual patients. The patients had a mean (standard deviation [SD]) age of 71.2 (14.2) years, and 34.4 % were female. In-hospital mortality was 82.1 %, and 24.7 % of patients were admitted to the Intensive Care Unit (ICU). The median (interquartile range [IQR]) time between observations was 232 (94 – 331) minutes, and the median (IQR) time from the most recent set of observations to an IHCA event was 112 (43–223) minutes. 94.9 % of observation sets occurring within 24 h of IHCA were complete.

**Fig. 1:**
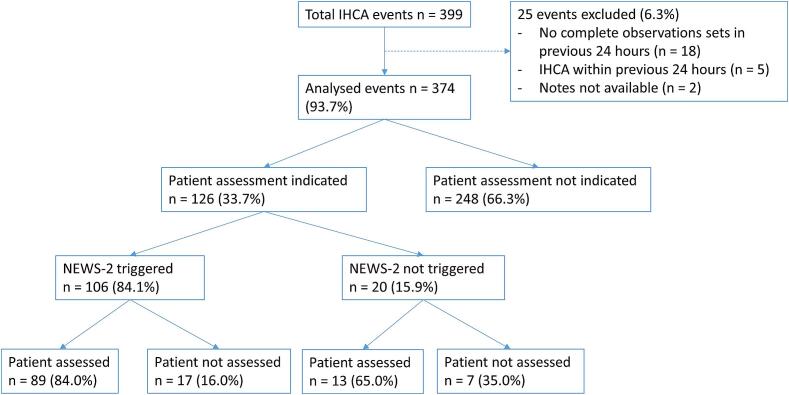
Flow chart of analysed in-hospital cardiac arrest events. IHCA = in-hospital cardiac arrest; NEWS-2 = National Early Warning Score-2.

Urgent patient assessment was deemed to be indicated in the lead-up to 126/374 (33.7 %) of IHCA cases (Fig. 1). NEWS-2 was triggered during the period of deterioration in 106/126 (84.1 %) of these cases. In 13 cases NEWS-2 was triggered solely through a single observation scoring 3 points, rather than a total score of ≥ 5. An urgent patient assessment took place in 102 out of 126 cases in which this was indicated (81.0 %), with this proportion being 89/106 (84.0 %) in cases where NEWS-2 was triggered, and 13/20 (65.0 %) in cases where NEWS-2 was not triggered (p = 0.048, Chi squared test).

**Fig. 2:**
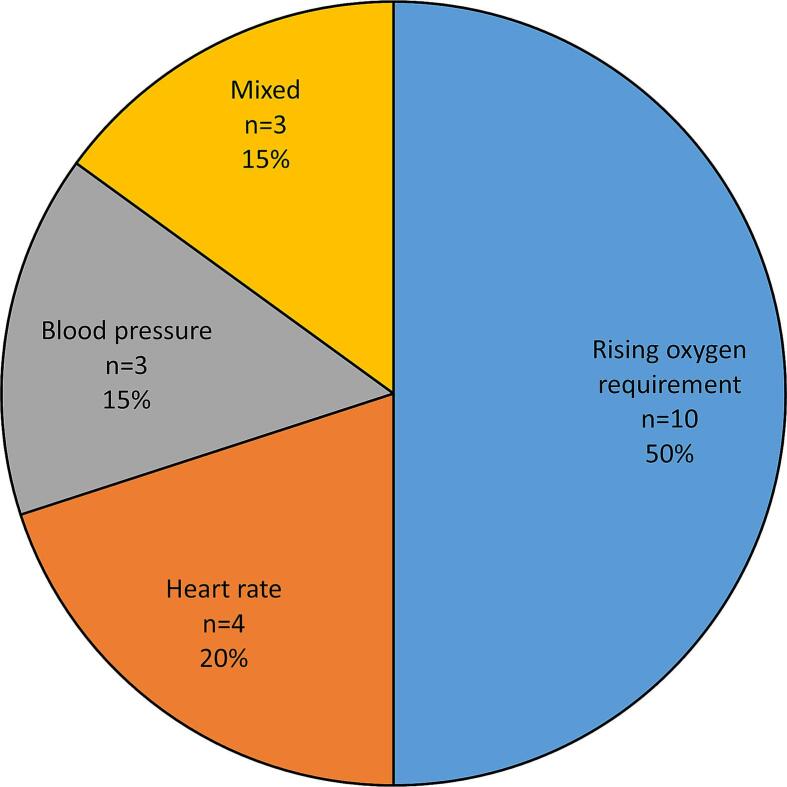
Vital sign abnormalities preceding in-hospital cardiac arrest in cases in which urgent patient assessment was indicated but NEWS-2 was not triggered.

Fig. 2 summarises the changes in observations which occurred prior to IHCA in the NEWS-2 miss cases. 10 out of 20 cases had predominant abnormalities in oxygenation, with this being manifested in all cases by a new or rising oxygen requirement rather than a drop in oxygen saturations. The remainder of the cases had heart rate, blood pressure or mixed abnormalities.

## Discussion

### Main findings

One third of IHCA cases were preceded by clinically important abnormalities in vital signs that warranted an urgent patient assessment, whereas two thirds arose suddenly, in some cases representing cardiac arrhythmias occurring in the context of acute coronary syndromes. Among cases in which an urgent patient assessment was indicated, NEWS-2 was triggered during the period of deterioration in 84 % of cases, with 10 % of the cases being detected solely by a single observation scoring 3 points, and not by a total NEWS-2 score of ≥ 5. We identified 24 cases in which an urgent assessment did not take place despite being indicated (failure-to-assess), as well as 20 cases in which NEWS-2 was not triggered despite an assessment being indicated (NEWS-2 miss). Failure-to-assess occurred more often if NEWS-2 was not triggered. While we cannot prove a causal link, it is possible that in some cases the failure of NEWS-2 to trigger led to a false sense of security among ward staff, resulting in an urgent patient assessment not being requested. Half of NEWS-2 miss cases were characterised by a new or rising oxygen requirement.

## Limitations

This was a retrospective study conducted in a single centre, and future studies should aim to replicate this work in a variety of different hospital settings. Clinical review of cases had an element of subjectivity and could have been subject to hindsight bias, given the reviewers’ knowledge of the eventual outcome. This was mitigated by each case being reviewed independently by three different clinicians. It was not within the scope of the study to assess whether the professional role of the person recording clinical observations had an impact on failure-to-assess. We were also not able to objectively assess nursing concern, which is known to be a significant predictor of patient deterioration^7^.

## Conclusions

A significant proportion of IHCA events are preceded by clinically important abnormalities in vital signs which are not detected by NEWS-2. Acute hospitals should implement systems to ensure that a new or rising oxygen requirement triggers an urgent patient assessment, and we suggest that this should be included in the next iteration of NEWS.

## CRediT authorship contribution statement

**Sherif Gonem:** Writing – original draft, Investigation, Formal analysis, Data curation, Conceptualization. **Daniella Draicchio:** Investigation. **Ayad Mohamed:** Investigation. **Sally Wood:** Writing – review & editing. **Kelly Shiel:** Resources, Data curation. **Steve Briggs:** Resources, Data curation. **Tricia M McKeever:** Writing – review & editing. **Dominick Shaw:** Writing – review & editing.

## Declaration of competing interest

The authors declare that they have no known competing financial interests or personal relationships that could have appeared to influence the work reported in this paper.

**Data Access Statement**

Study data are not currently available for sharing due to ethical and institutional restrictions. Requests for data sharing should be directed to the corresponding author.
